# Pregnancy With Massive Splenomegaly and Pancytopenia: A Case Report

**DOI:** 10.7759/cureus.50656

**Published:** 2023-12-17

**Authors:** Rajasi K Sengupta, Saunitra A Inamdar

**Affiliations:** 1 Obstetrics and Gynaecology, Datta Meghe Medical College, Datta Meghe Institute of Higher Education and Research (Deemed to be University), Wardha, IND

**Keywords:** hypersplenism, case report, pancytopenia, splenomegaly, pregnancy

## Abstract

Massive splenomegaly complicating pregnancy is a rare clinical entity that poses special difficulties, such as anemia, thrombocytopenia, ascites, and jaundice. This case report of a pregnant woman with large splenomegaly and pancytopenia highlights the value of prompt diagnosis and effective treatment. Splenomegaly can have a number of causes, including viral infections, hematological problems, portal hypertension, and metabolic abnormalities. A 29-year-old gravida 3 woman at 37 weeks of gestation who had massive splenomegaly was admitted and underwent a cesarean section to avoid complications of splenomegaly. The case report discusses the difficulties in obstetric management caused by enormous splenomegaly during pregnancy, including the choice of delivery method. Significant complications include splenic rupture and bleeding, particularly when pancytopenia is present. The need for several transfusions, the potential side effects of transfusion therapy, and factors related to the origin of splenomegaly when assessing maternal-fetal outcomes are discussed in this case report. The study concludes that in cases with pancytopenia splenomegaly during pregnancy, vigilant monitoring, prompt intervention, and a multidisciplinary approach are crucial to achieve positive outcomes for both the mother and the fetus.

## Introduction

Splenomegaly is characterized by enlargement of the spleen, distinct from hypersplenism, denoting an excessively active spleen. This condition constitutes a nonspecific clinical manifestation frequently associated with a variety of underlying etiologies, including infectious diseases such as malaria, leishmania, and schistosomiasis, hematological disorders such as sickle cell disease and hemoglobinopathy, infiltration of the spleen by malignant neoplasms, portal hypertension, immunological dysfunction, liver cirrhosis, metabolic disorders including amyloidosis and Gaucher disease, and extramedullary hematopoiesis [1]. The spleen, a crucial lymphoid organ in the human body, typically measures approximately 1x3x5 inches and is placed between the 9th and 11th ribs. However, during pregnancy, the appearance of massive splenomegaly is a relatively rare condition, and its impact can be exacerbated by concurrent complications such as anemia, thrombocytopenia, ascites, and jaundice [2]. In this report, we present the case of a pregnant patient who had massive splenomegaly, for which the etiology remains undefined and is further complicated by the presence of pancytopenia. This case underscores the importance of a complete understanding of splenic disorders during pregnancy, especially when accompanied by enigmatic clinical presentations, and the need for timely and effective medical intervention [3].

## Case presentation

A 29-year-old woman, a third gravida, presented at the hospital in a rural region of central India with a history of vaginal leakage that persists for 3 hours. She was in full term in her pregnancy, experienced mild abdominal discomfort, and exhibited signs of early labor. The patient had been receiving prenatal care at our hospital since the early stages of her pregnancy. She had previously suffered two abortions, both occurring at approximately 6 weeks of gestation.

**Figure 1:**
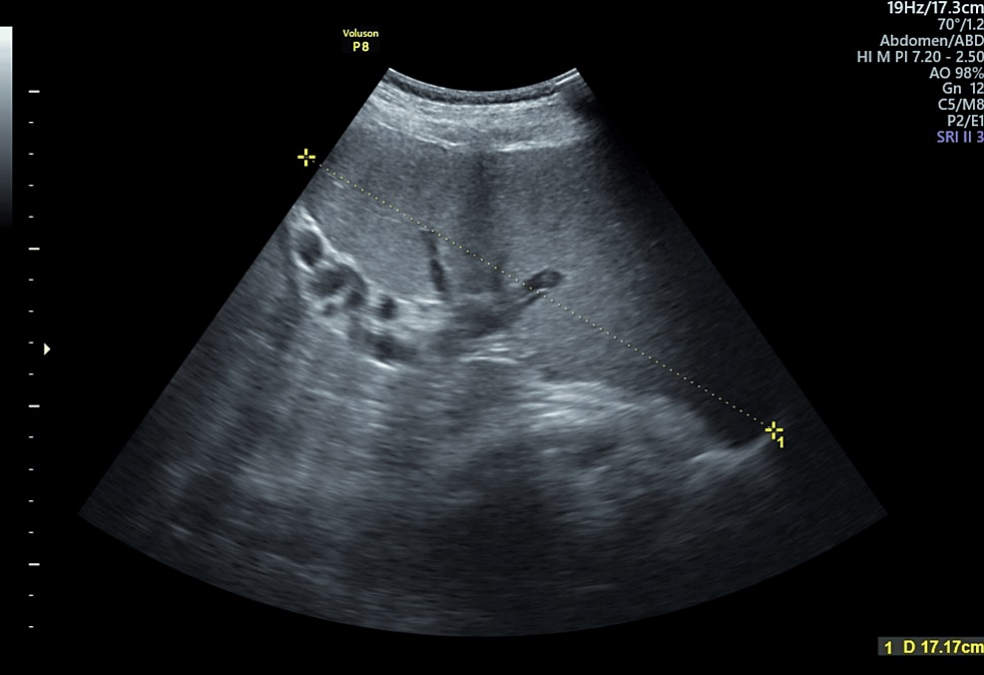
Ultrasonography revealing the presence of splenomegaly Yellow line indicates the length of the spleen

During the seventh month of her pregnancy, ultrasonography revealed the presence of splenomegaly, with her spleen measuring 17.17 cm in length (Figure 1). Ultrasound also identified splenic hilar and lienorenal varicosities, as well as a moderate level of oligohydramnios, with an amniotic fluid index of 6.0 cm.

The patient has a longstanding history of mild bronchial asthma dating back to childhood; however, she is presently not undergoing regular medication for this condition. Previously, the patient had been admitted to the hospital twice due to anemia, specifically nutritional anemia with a normocytic normochromic smear, necessitating blood transfusions on both occasions. Notably, laboratory investigations, including liver function tests and coagulation profile, conducted during the antepartum period revealed normal results. Furthermore, both the sickling test (solubility test) and the Plasmodium falciparum test yielded negative results. At 35 weeks of gestational age, the patient was admitted for pancytopenia. Following transfusion, her hemoglobin (Hb) level reached 11.1 gm%, with a total leukocyte count of 5,900 cubic millimeters and a platelet count of 100,000 per cubic millimeter (Table 1).

**Table 1:** Laboratory investigations LFT, liver function test; ALT, alanine transaminase; AST, aspartate transaminase; ALP, alkaline phosphatase; KFT, kidney function test; BUN, blood urea nitrogen; Na, sodium; K, potassium; CBC, complete blood count; Hb, hemoglobin; WBC, white blood cells; RBC, red blood cells; PT, prothrombin time; INR, international normalized ratio; APTT, activated partial thromboplastin time

Test	Observed Value	Reference Range
LFT		
Total bilirubin	0.8 mg/dL	0.3-1.0 mg/dL
Direct bilirubin	0.1 mg/dL	0.0-0.3 mg/dL
Indirect bilirubin	0.2 mg/dL	0.1-0.7 mg/dL
ALT	23U/L	7-56 U/L
AST	17 U/L	5-40 U/L
ALP	107 U/L	44-147 U/L
Total protein	5.8 g/dL	6.0-8.3 g/dL
Albumin	3.0 g/dL	3.5-5.5 g/dL
KFT		
Blood urea nitrogen	11.8 mg/dL	7-20 mg/dL
Serum creatinine	0.8 mg/dL	0.6-1.3 mg/dL
Na	142 mmol/L	135-145 mmol/L
K	4.2 mmol/L	3.5-5.0 mmol/L
CBC		
Hemoglobin	9.2 g/dL	12.1-15.1 g/dL
WBC	3.2 x 10^3^/µL	4.0-11.0 x 10^3^/µL
Platelets	100 x 10^3^/µL	150-450 x 10^3^/µL
RBC	4.0 x 10^3^/µL	4.0-5.0 x 10^6^/µL
Coagulation profile		
PT	11 seconds	10.0-14.0 seconds
INR	1.1	0.8-1.2
APTT	27 seconds	25-35 seconds

Due to cephalopelvic disproportion in labor and the presence of massive splenomegaly with enlarged splenic varices, the patient underwent an emergency lower segment cesarean section. A full-term male neonate, weighing 3.47 kg, was delivered in the vertex position. The amniotic fluid was thin and meconium tinged. The baby had two loops of cord wrapped around the neck, but responded well and cried immediately after birth. Given the high-risk nature of the case, the patient was transferred to the intensive care unit (ICU).

On the second postoperative day, her Hb level dropped to 6.6 gm% and her platelet count decreased to 91,000 per cubic millimeter. The patient also experienced abdominal distention. A surgical evaluation was inconclusive, leading to the decision to withhold oral intake and administer two units of packed red cells, two units of fresh frozen plasma, and one unit of platelet concentrate. However, the patient and her family refused any further transfusions. Subsequently, she developed a mild pleural effusion on the left side and exhibited mild hepatomegaly. A medical assessment was performed, leading to the initiation of a treatment regimen involving a combination of 50 mg and frusemide 20 mg once a day. Furthermore, 15 mL of syrup lactulose at bedtime was advised to maintain a high-fiber diet to address constipation.

On the third postoperative day, her abdominal distension gradually subsided, allowing her to be transferred from the ICU to the general ward. The surgical stitches were removed on the 12th day after the operation, and the remaining postoperative period was uneventful.

## Discussion

Splenomegaly with pancytopenia is a rare condition during pregnancy that can be challenging to diagnose and manage. Several studies have reported cases of pancytopenia-related splenomegaly in pregnancy, highlighting the need for a planned and timely diagnosis and management planned to ensure good maternal and fetal outcomes. Although there is little literature on massive splenomegaly and its outcome during pregnancy, massive splenomegaly has been observed to be associated with adverse obstetric outcomes.

Enlargement of the spleen, known as splenomegaly, is an indiscriminate clinical manifestation commonly associated with various medical conditions. This enlargement is frequently observed in the context of infectious diseases such as malaria, leishmania, and schistosomiasis, as well as hematological disorders such as sickle cell disease and hemoglobinopathy. Furthermore, splenomegaly may arise from cancer infiltrating the spleen, portal hypertension, immunological abnormalities, liver cirrhosis, and metabolic disorders including amyloidosis and Gaucher disease. Furthermore, extramedullary hematopoiesis is also implicated in the etiology of splenomegaly [4,5].

Hyperreactive malarial splenomegaly, previously identified as tropical splenomegaly syndrome or big spleen disease, represents a prevalent etiology of substantial splenomegaly within regions characterized by endemic malaria [6]. In the regions of Asia and Africa, tropical splenomegaly occurs predominantly as a consequence of infections attributable to malaria, sickle cell disease, and schistosomiasis [2]. Enlargement of the spleen during gestation poses an additional diagnostic challenge for obstetricians. Identification of this condition, particularly in the later stages of pregnancy, is challenging through conventional clinical assessment. The decision regarding the mode of delivery in such cases is important and poses a dilemma for the obstetrician. Normal labor and delivery can be associated with spontaneous splenic rupture and massive hemoperitoneum, which requires emergency surgery. During a cesarean section, the spleen can rupture while delivering the baby, creating a life-threatening emergency that must be tackled immediately [7]. All these concerns pose a challenge in the obstetric management of cases of massive splenomegaly.

Added to the size of the spleen, if there is pancytopenia, the risk of bleeding increases. Thrombocytopenia and anemia are both independent risk factors for obstetric hemorrhage. The need for multiple transfusions during pregnancy and intrapartum increases. The inherent problems related to multiple transfusions, such as fluid overload, patient compliance, infections, and even financial burden on the couple must be taken into consideration when planning the treatment of pregnancy cases with splenomegaly and thrombocytopenia.

The cause of splenomegaly is also important in deciding the maternal-fetal outcome. Infectious diseases such as malaria and tuberculosis affect the mother and fetus. Idiopathic thrombocytopenia and portal hypertension have a higher risk of hemorrhage and complications in the mother.

In a study by Ali and Eldin [1] in Sudan, the etiological factors that contribute to splenomegaly were delineated. The identified causes included tropical splenomegaly (52.7%), portal hypertension (26.3%), tuberculosis (8.8%), leishmaniosis (5.3%), idiopathic thrombocytopenia (3.5%), and cases with an unidentified cause (3.5%). The prevalence of massive splenomegaly was observed to be the highest, accounting for 50.9%, followed by mild (31.6%) and moderate (17.5%) presentations. It should be noted that none of the evaluated patients required splenectomy. These findings offer valuable information on the distribution and severity of cases of splenomegaly within the studied population [7]. Reports of obstetric complications included instances of intrauterine growth restriction (19.3%), preterm labor (17.5%), miscarriage (12.3%), and stillbirth (3.5%). In particular, respiratory distress syndrome, transient tachypnea of the newborn, and stillbirth were identified in 18%, 6%, and 4% of cases, respectively. It should be noted that a higher incidence of adverse obstetric outcomes was observed among individuals with massive splenomegaly compared to other categorizations [7].

Cautionary remarks are warranted regarding the rare, yet potentially life-threatening clinical occurrence of splenic rupture. The spontaneous rupture of the spleen during pregnancy is a rare event, with a predilection for manifestation in the third trimester or the postpartum period. The pathogenesis of splenic rupture in this context has been postulated to involve factors such as splenic enlargement, increased blood volume, and diminished peritoneal cavity space, all of which are associated with physiological changes occurring during pregnancy [8]. Identifying splenic rupture during pregnancy presents a diagnostic challenge due to the overlap in clinical manifestations with other conditions such as uterine rupture and placental abruption. The established medical approach to the management of spontaneous splenic rupture involves the implementation of splenectomy. Maternal mortality is often attributed to extensive hemorrhaging, concurrent hemorrhagic shock, and consumptive coagulopathy. Hemodynamic decompensation in the maternal system can precipitate a sudden decline in uteroplacental perfusion, resulting in the manifestation of "fetal distress" and eventually fetal demise [8].

## Conclusions

In conclusion, splenomegaly with pancytopenia during pregnancy can be challenging to diagnose and manage. It is important to diagnose the etiology of both splenomegaly and pancytopenia. There is always a need for careful monitoring of maternal and fetal health during pregnancy and the importance of timely intervention to manage complications. A multidisciplinary approach is necessary to manage the condition, which was used effectively in our patient. This helped to obtain a favorable outcome for both the mother and the baby in our case. In all high-risk obstetric cases, timely diagnosis, appropriate treatment, and careful follow-up are essential to ensure good maternal and fetal outcomes. We must remember that we deal with two lives, and thus the approach has to be tailored to suit these conditions.
